# LESSONS FROM FIVE YEARS OF POLLING THROUGH THE NATIONAL POLL ON HEALTHY AGING

**DOI:** 10.1093/geroni/igac059.903

**Published:** 2022-12-20

**Authors:** Erica Solway, Teresa Keenan, Cheryl Lampkin, Matthias Kirch, Dianne Singer, Jeffrey Kullgren, Preeti Malani

**Affiliations:** University of Michigan, Ann Arbor, Michigan, United States; AARP, Washington, District of Columbia, United States; AARP, Washington, District of Columbia, United States; University of Michigan, Ann Arbor, Michigan, United States; University of Michigan, Ann Arbor, Michigan, United States; University of Michigan, Ann Arbor, Michigan, United States; University of Michigan, Ann Arbor, Michigan, United States

## Abstract

Since 2017, the National Poll on Healthy Aging (NPHA) has surveyed older adults on a vast array of topics related to health, health care and health policy. With robust nationally representative samples of 2,000 adults age 50-80, the poll’s format allows for the fielding of timely questions with important public health and policy implications. For example, polls examining delayed care during the COVID-19 pandemic and experiences of physical deconditioning and falls helped to demonstrate the ways in which the pandemic may have long-lasting effects if strategies are not implemented to address them. In this presentation we will discuss how the NPHA has increased awareness of the facilitators and barriers to healthy aging and where gaps exist in supporting older adults to live with optimal health and well-being. This presentation will also describe how researchers can access publicly available NPHA data to inspire their own future research.

